# Tumor microenvironment dictates regulatory T cell phenotype: Upregulated immune checkpoints reinforce suppressive function

**DOI:** 10.1186/s40425-019-0785-8

**Published:** 2019-12-04

**Authors:** Hye Ryun Kim, Hyo Jin Park, Jimin Son, Jin Gu Lee, Kyung Young Chung, Nam Hoon Cho, Hyo Sup Shim, Seyeon Park, Gamin Kim, Hong In Yoon, Hyun Gyung Kim, Yong Woo Jung, Byoung Chul Cho, Seong Yong Park, Sun Young Rha, Sang-Jun Ha

**Affiliations:** 1 Yonsei Cancer Center, Division of Medical Oncology, Yonsei University College of Medicine, 50 Yonsei-ro, Seodaemun-Gu, Seoul, 120-752 South Korea; 2 Department of Biochemistry, College of Life Science & Biotechnology, Yonsei University, Seoul, South Korea; 3 Department of Thoracic and Cardiovascular Surgery, Yonsei University College of Medicine, 50 Yonsei-ro, Seodaemun-Gu, Seoul, 120-752 South Korea; 4 Department of Pathology, Yonsei University College of Medicine, Seoul, South Korea; 5 Department of Radiation Oncology, Yonsei University College of Medicine, Seoul, South Korea; 6 Department of Pharmacy, Korea University, Sejong, South Korea

**Keywords:** Tumor microenvironment, Regulatory T cells, Immune checkpoints, Programmed cell death 1 receptor

## Abstract

**Background:**

Regulatory T (T_reg_) cells have an immunosuppressive function in cancer, but the underlying mechanism of immunosuppression in the tumor microenvironment (TME) is unclear.

**Methods:**

We compared the phenotypes of T cell subsets, including T_reg_ cells, obtained from peripheral blood, malignant effusion, and tumors of 103 cancer patients. Our primary focus was on the expression of immune checkpoint (IC)-molecules, such as programmed death (PD)-1, T-cell immunoglobulin and mucin-domain containing (TIM)-3, T cell Ig and ITIM domain (TIGIT), and cytotoxic T lymphocyte antigen (CTLA)-4, on T_reg_ cells in paired lymphocytes from blood, peritumoral tissue, and tumors of 12 patients with lung cancer. To identify the immunosuppressive mechanisms acting on tumor-infiltrating T_reg_ cells, we conducted immunosuppressive functional assays in a mouse model.

**Results:**

CD8^+^, CD4^+^ T cells, and T_reg_ cells exhibited a gradual upregulation of IC-molecules the closer they were to the tumor. Interestingly, PD-1 expression was more prominent in T_reg_ cells than in conventional T (T_conv_) cells. In lung cancer patients_,_ higher levels of IC-molecules were expressed on T_reg_ cells than on T_conv_ cells, and T_reg_ cells were also more enriched in the tumor than in the peri-tumor and blood. In a mouse lung cancer model, IC-molecules were also preferentially upregulated on T_reg_ cells, compared to T_conv_ cells. PD-1 showed the greatest increase on most cell types, especially T_reg_ cells, and this increase occurred gradually over time after the cells entered the TME. PD-1 high-expressing tumor-infiltrating T_reg_ cells displayed potent suppressive activity, which could be partially inhibited with a blocking anti-PD-1 antibody.

**Conclusions:**

We demonstrate that the TME confers a suppressive function on T_reg_ cells by upregulating IC-molecule expression. Targeting IC-molecules, including PD-1, on T_reg_ cells may be effective for cancer treatment.

## Background

The recent development of immune checkpoint inhibitors (ICIs) has revolutionized cancer treatment. ICIs specific for anti-cytotoxic T lymphocyte antigen (CTLA)-4 or anti-programmed death (PD)-1 have improved patient survival and have been approved for the treatment of several cancer types, including non-small cell lung cancer (NSCLC), melanoma, head and neck cancer, bladder cancer, and renal cell cancer [1–3].

The tumor microenvironment (TME) and the immune system play critical roles in cancer progression and clinical outcome [4, 5]. Regulatory T (T_reg_) cells are highly immunosuppressive and contribute to the maintenance of self-tolerance and immune homeostasis in humans [6, 7]. T_reg_ cells infiltrate tumors and promote their progression by suppressing antitumor immunity in the TME. Depleting T_reg_ cells can lead to spontaneous tumor regression due to enhanced antitumor response [7, 8]. Interaction of T_reg_ cells with TME enhances their immunosuppressive function and proliferative capacity. Several studies have shown that tumor-infiltrating T_reg_ cells are phenotypically distinct from those in peripheral blood (PB) and normal tissues [9, 10], suggesting that their immunosuppressive function depends on environmental factors.

T_reg_ cells suppressive functions are associated with the expression of several immune checkpoint molecules (ICs), such as PD-1, CTLA-4, T-cell immunoglobulin and mucin-domain containing-3 (TIM)-3, and T cell Ig and ITIM domain (TIGIT) [3, 6, 11–14]. CTLA-4 and TIGIT act as tumor suppressors and thus, modulate the immune response in the TME [6, 15, 16]. Although the PD-1/PD ligand (PD-L)1 interaction was shown to promote the conversion of conventional T (T_conv_) cells into T_reg_ cells to maintain the latter’s population [17–19], it remains controversial whether PD-1 expression by T_reg_ cells suppresses antigen-specific T cell immune responses [20–22].

Recent studies have reported that IC-molecules are upregulated on T_reg_ cells within the TME or upon chronic infection and that T_reg_ cells-mediated immunosuppression correlates with the expression of IC-molecules on these cells [6, 12]. The upregulation of these molecules has also been linked to tumor progression, as it likely reinforces the suppressive function of T_reg_ cells in the TME. We previously reported that an increased level of PD-1 on T_reg_ cells during chronic viral infection enhances CD8^+^ T cell immune suppression via interaction with PD-L1 on CD8^+^ T cells [12]. On the contrary, high PD-1 expression on T_reg_ cells indicates dysfunctional and exhausted IFN-γ-secreting T_reg_ cells that are enriched in tumor infiltrates and have possibly lost their suppressive function [23]. So far, the precise role of PD-1 in the function of tumor-infiltrating T_reg_ cells in the TME is controversial. Given the significance of PD-1 in modulating immune responses and its paradoxical role as both an activation and exhaustion marker, clarifying the function of PD-1-positive T_reg_ cells and their role in regulating anti-tumor immune responses is important [23].

To evaluate the suppressive function of tumor-infiltrating T_reg_ cells in the TME, we comprehensively compared the phenotypes of T cell subsets, including T_reg_ cells, obtained from PB, malignant effusion (ME), and tumor (TM) samples of patients with cancer. We also characterized T_reg_ cells in paired lymphocyte samples obtained from blood, peri-tumoral tissue, and tumors of patients with lung cancer. Using a lung cancer mouse model, we investigated the suppressive function and mechanism of action of tumor-infiltrating T_reg_ cells in the TME. We found that PD-1 was upregulated in tumor-infiltrating T_reg_ cells and played a role in suppressing CD8^+^ T cell proliferation through PD-1/PD-L1 interactions. These results suggest that infiltrated PD-1-expressing T_reg_ cells in TME are a potential therapeutic target for anti-cancer treatment.

## Methods

### Study design

Patients with stage IV cancer with ME and patients with cancer who planned to undergo surgical resection between April 2012 and December 2017 at the Severance Hospital were prospectively enrolled. Inclusion criteria were as follows: 1) over 20 years old; 2) stage IV cancer with malignant pleural effusion or ascites confirmed by cytology, or cancer with scheduled surgery; and 3) written informed consent. We collected 300 cc of effusions and simultaneously obtained 10 cc of PB from patients with stage IV cancer with ME, if available. In patients who underwent surgery, we collected TM-adjacent normal tissue, and 10 cc of PB to isolate peripheral blood lymphocytes (PBLs). The study was approved by the Institutional Review Board of Severance Hospital. We categorized the samples into three groups: 1) PBLs, 2) ME from patients with stage IV cancer, and 3) TM from patients with cancer who underwent surgery. To analyze the characteristics of T_reg_ cells in TME, we also collected paired peritumoral tissue lymphocytes (pTILs), tumor-infiltrating lymphocytes (TILs), and PBLs at the same day from 12 patients with NSCLC who underwent curative resection.

### Isolation of PB mononuclear cells and ME lymphocytes

PB mononuclear cells were isolated from 10 cc PB collected into EDTA tubes by separation over a Percoll (Sigma-Aldrich) gradient. Lymphocytes were isolated from 500 cc of ME by discontinuous density gradient centrifugation on Percoll. To isolate TILs, lung TMs were chopped and then incubated with a solution containing 1 mg/mL collagenase type IV (Worthington Biochemical) and 0.01 mg/mL DNaseI (Sigma-Aldrich) at 37 °C for 25 min. TILs were isolated by Percoll gradient after washing dissociated tissues with ice-cold RPMI1640.

### Flow cytometry and antibodies

Flow cytometry was performed using a FACS CANTOII (BD Biosciences, Franklin Lakes, NJ, USA) and CytoFLEX (Beckman Coulter, IN, USA). Data were analyzed using FlowJo software (Tree Star, OR, USA).

For immunolabeling of human samples, fluorophore-conjugated monoclonal antibodies against the following proteins were used: CD4 (RPA-T4), CD3 (OKT3), PD-1 (EH12.2H7), and CTLA-4 (BNI3) (all from Biolegend, San Diego, CA, USA); TIM-3 (344823) and TIGIT (741182) (both from R & D Systems, Minneapolis, MN, USA); CD25 (M-A251) (BD Biosciences, Franklin Lakes, NJ, USA); and Foxp3 (PCH101) (eBioscience, San Diego, CA, USA). The LIVE/DEAD Fixable Red Dead Cell Stain kit was from Invitrogen (Carlsbad, CA, USA). T_reg_ cells labeled with various antibodies (except for the antibody against Foxp3) were fixed and permeabilized with Foxp3 fixation/permeabilization solution (eBioscience). Foxp3 antibody was then administered for intracellular labeling of T_reg_ cells. The proportion of CD4^+^ and CD8^+^ T cells among total lymphocytes was determined, and the fraction of Foxp3-positive CD4^+^ T cells was quantified.

For immunolabeling of mouse samples, fluorophore-conjugated monoclonal antibodies against the following proteins were used: CD4 (RM4–5), Ly5.1 (A20), PD-1 (29F.1A12), TIM-3 (RMT3–23), NK1.1 (PK136), and DX5 (DX5) (all from Biolegend); and CD8 (53-6.7), CD25 (PC61.5), CTLA-4 (UC10-4B9), TIGIT (G1GD7), and F4/80 (BM8) (all from eBioscience); and CD11b (M1/79) (BD Biosciences). The LIVE/DEAD Fixable Near-IR Dead Cell Stain kit was from Invitrogen. T_reg_ cells labeled with various antibodies (except for the antibodies against Foxp3 and CTLA-4) were fixed and permeabilized with Foxp3 fixation/permeabilization solution (eBioscience, San Diego, CA, USA). Foxp3 antibody was then administered for intracellular labeling of T_reg_ cells. The proportions of CD4^+^ and CD8^+^ T cells among lymphocytes were determined, and the fraction of Foxp3-positive CD4^+^ T cells was quantified. To prevent myeloid cells from non-specific staining, samples were preincubated with anti-CD16/32 (93) (eBioscience) before immunolabeling with fluorophore-conjugated antibodies.

### Mouse TM model and lymphocyte isolation

Female C57BL/6, C57BL/6-Rag2^−/−^, and C57BL/6-Ly5.1 congenic mice (5–6 weeks) were purchased from Charles River Laboratories (Wilmington, MA, USA) and Jackson Laboratories (Bar Harbor, ME, USA). To generate lung TM bearing mice, 5 × 10^5^ TC-1 cells were intravenously injected into C57BL/6 mice via the tail-vein. Mice were sacrificed on day 21 post-injection. Lymphocytes were isolated from the spleen, normal lung, and lung tumor as previously described [9]. The number of tumor nodules on left upper lobe of the lung was counted at day 12, 16, and 21 post-injection. All animal protocols were approved by the Institutional Animal Care and Use Committee of the Yonsei University Laboratory Animal Research Center (2013–0115).

### In vitro suppression assay using mouse lymphocytes

For the T_reg_ cells suppression assay, CD4^+^CD25^+^ T_reg_ cells (10^5^/well) were co-cultured with CD8^+^ T cells (10^5^/well) with Dynabeads mouse T-activator CD3/CD28 (Thermo Fisher Scientific, Waltham, MA, USA) in a 96-well U-bottom plate at 37 °C for 72 h. For the CellTrace Violet dilution assay, CD8^+^ T cells were isolated from the spleen of naïve mice using a CD8^+^ T Cell Isolation kit (Miltenyi Biotec, Bergisch Gladbach, Germany) and labeled with 5 μM CellTrace Violet (Thermo Fisher Scientific). CD4^+^CD25^+^ T_reg_ cells were separately isolated from the spleen and tumor of TM-bearing mice on day 21 post-injection using CD4^+^CD25^+^ Regulatory T Cell Isolation kit (Miltenyi Biotec, Bergisch Gladbach, Germany). To inhibit cell migration, Transwell membranes (0.4 mm pore; BD Biosciences) were inserted into 24-well plate. CD4^+^CD25^+^ T_reg_ cells (10^6^/well) were co-cultured with CD8^+^ T cells (10^6^/well) with Dynabeads mouse T-activator CD3/CD28 (Thermo Fisher Scientific) in a 24-well plate at 37 °C for 72 h.

For PD-1 blockade in tumor-infiltrating T_reg_, CD4^+^CD25^+^ T_reg_ cells (2.5 × 10^4^/well) isolated from tumor lymphocytes of TM bearing mice on day 14 post-injection were preincubated with 10 μg/mL anti-PD-1 antibody (RMP1–14) or rat IgG2a isotype control (2A3) (Bio X Cell) at 4 °C for 1 h, washed twice, and then co-cultured with CD8^+^ T cells (10^5^/well) in the presence of mouse T-activator CD3/CD28 Dynabeads for 68 h.

### Adoptive cell transfer for in vivo suppression assay

To examine the functionality of TIL T_reg_ (PD-1^hi^) and spleen T_reg_ (PD-1^lo^) cells, CD4^+^CD25^+^ T_reg_ cells were isolated from the tumor and spleen of TM bearing mice at day 21 post-injection using CD4^+^CD25^+^ Regulatory T Cell Isolation kit (Miltenyi Biotec). Ly5.1^+^ CD8^+^ T cells were isolated from naïve C57BL/6-Ly5.1 congenic mice. Ly5.1^+^ CD8^+^ T cells (2 × 10^6^) were injected i.v. into recipient Rag2^−/−^ mice alone or with Ly5.2^+^ TIL T_reg_ or spleen T_reg_ (1 × 10^6^). At day 7 after cell transfer, splenocytes isolated from Rag2^−/−^ mice were analyzed for homeostatic expansion of the Ly5.1^+^ CD8^+^ T cell population using FACS.

### In vitro suppression assay using human lymphocytes

CD4^+^CD25^+^ T_reg_ cells were isolated from the tumor tissue and peripheral blood of NSCLC patients, using human CD4^+^CD25^+^CD127^dim/−^ Regulatory T Cells Isolation Kit II (Miltenyi Biotec, Bergisch Gladbach, Germany). CD8^+^ T cells were isolated from the paired peripheral blood of NSCLC patients using human CD8^+^ T Cell Isolation kit (Miltenyi Biotec, Bergisch Gladbach, Germany) and subsequently labeled with 5 μM CellTrace Violet. The CD8^+^ T cells (10^5^/well) were co-cultured with CD4^+^CD25^+^ T_reg_ cells (5 × 10^4^/well) isolated from either tumor tissue or peripheral blood in the presence of 2.5 μl/well of Dynabeads human T-activator CD3/28 (Thermo Fisher Scientific) at 37 °C for 72 h.

### Multi-color immunofluorescence analysis

For multicolor immunofluorescence analysis, lungs were isolated, fixed with 2% paraformaldehyde/phosphate buffered saline overnight at 4 °C, and then embedded in OCT compound (Sakura). Tissue blocks were frozen in 2-methyl butane and cooled on dry ice. Frozen blocks were cut to a thickness of 8 μm and mounted on the silane-coated slide. Sections were stained with 4,6-diamidino-2-phenylindole (DAPI; Invitrogen) and with antibodies for anti-CD8α (Clone 53–6.7), anti-CD4 (clone RM4–5), anti-CD279 (clone RMP1–30), and anti-GFP (clone 1GFP63) for amplification of Foxp3-GFP signals (Biolegend). Streptavidin-conjugated horseradish peroxidase was used for staining of biotin-conjugated antibodies, and TSA Cyanine 3 Tyramidetetramethylrhodamine reagent (SAT704A001EA; PerkinElmer) was subsequently added for amplification. Images was acquired using a microscope (Carl Zeiss Co. Ltd) and analyzed with ImageJ 1.50b software.

### Statistical analysis

Data were analyzed using Prism 5.0 software (GraphPad Inc., CA, USA). The Student’s *t*-test, one-way analysis of variance, and the least significant difference test were used, where appropriate, to evaluate the significance of differences among groups. All statistical analyses were conducted with a significance level of α = 0.05 (*P* < 0.05).

## Results

### Patient characteristics

**Fig. 1:**
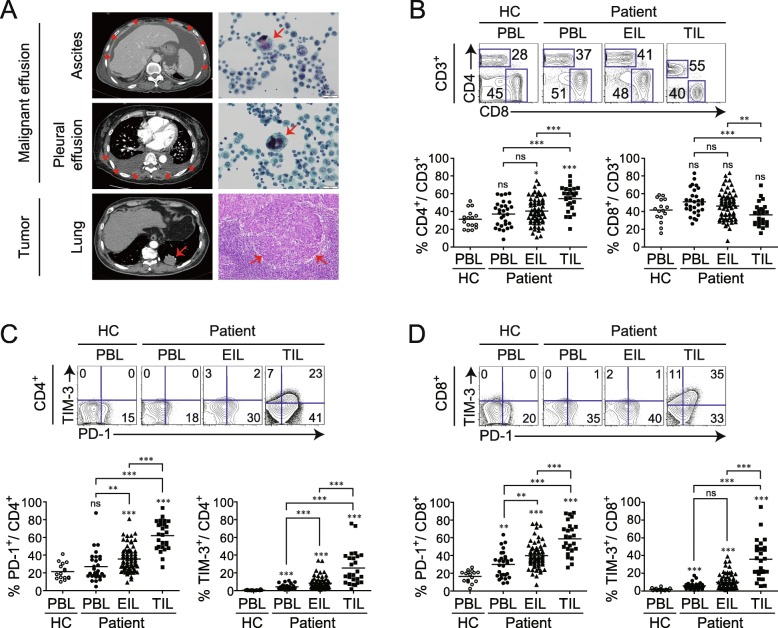
T cell characteristics and PD-1 expression in T_conv_ of patients with cancer. Malignant effusions, such as ascites, and pleural effusions were extracted from patients with stage IV cancer. Tumor-infiltrating lymphocytes were obtained from the tumors of patients with non-small cell lung cancer (NSCLC) and colon cancer. **a** Left, computed tomography images showing malignant ascites (upper), pleural effusion (middle), and lung cancer lesions in left lower lobe (bottom) of a patient with NSCLC. Right, cytological analysis of malignant effusion and histological analysis of lung cancer tissue. Red arrows indicate malignant effusion and cancer (left column) and tumor cells (right column). **b** Proportions of CD4^+^ and CD8^+^ T cells among CD3^+^ T cells. Representative plots (upper) and statistics (bottom) are shown. **c**, **d** PD-1 and TIM-3 expression on CD4^+^ and CD8^+^ T cells. Representative plots of PD-1 and TIM-3 expression (upper) and percentages of total PD-1^+^ (bottom left) and TIM-3^+^ (bottom right) cells among CD4^+^ and CD8^+^ T cells are shown. Peripheral blood lymphocytes (PBLs), effusion-infiltrating lymphocytes (EILs), and tumor-infiltrating lymphocytes (TILs) were isolated from healthy control donors (HC) and patients with cancer (PBLs of HC, *n* = 16; PBLs, *n* = 28; EILs, *n* = 76; TILs, *n* = 31). Lines in the scatterplot represent the mean values. ns, not significant; *P < 0.05, **P < 0.01, ***P < 0.001 (Mann-Whitney test)

We enrolled 103 patients: 72 were stage IV cancer patients with ME, and 31 were patients with operable disease (not stage IV) who underwent surgical resection. Detailed information of the patients from which PB, ME, or TM were obtained is described in Additional file 6: Table S1. The total number of tumor specimens was divided into three groups based on the specimen type: PB, 20.7% (28/135); ME, 56.2% (76/135); and TM, 23.1% (31/135). Detailed analyses of immune subsets as well as the levels of their immune checkpoints were performed in PBLs (PB group), effusion-infiltrating lymphocytes (EILs) (ME group), and TILs (TM group). Primary cancer types in the ME group were NSCLC, 43.1% (31/72); gastric cancer, 22.2% (16/72); colon cancer, 5.6% (4/72), and breast cancer, 5.6% (4/72). The types of ME were ascites, 59.7% (43/76) and pleural effusion, 45.8% (33/76), with four patients having both (Additional file 6: Table S1). The presence of malignant cancer cells and TILs in TM or ME was pathologically or cytologically confirmed (Fig. 1a).

### T_conv_ with exhausted phenotypes are abundant in TM and ME

To investigate T cell subsets in three different tumor specimens, we compared the ratio of CD4^+^ and CD8^+^ T cells in PBLs, EILs, and TILs isolated from PB, ME, and TM, respectively. The percentage of CD4^+^ T cells was higher among TILs than among PBLs or EILs. In contrast, the percentage of CD8^+^ T cells was markedly lower among TILs than among PBLs or EILs (Fig. 1b), suggesting that the migration of cytotoxic lymphocytes (CTLs) into the TM was inhibited.

The phenotypes of PBLs, EILs, and TILs were compared by quantifying CD4^+^ and CD8^+^ T cells expressing PD-1 and TIM-3. The percentage of PD-1- or TIM-3-expressing CD4^+^ or CD8^+^ T cells was the highest among TILs, with lower percentages in EILs and PBLs (Fig. 1c, d), suggesting that T cells derived from TM and ME show more pronounced T cell exhaustion than those derived from PBLs.

### High expression of PD-1 in T_reg_ cells of ME and TM

**Fig. 2:**
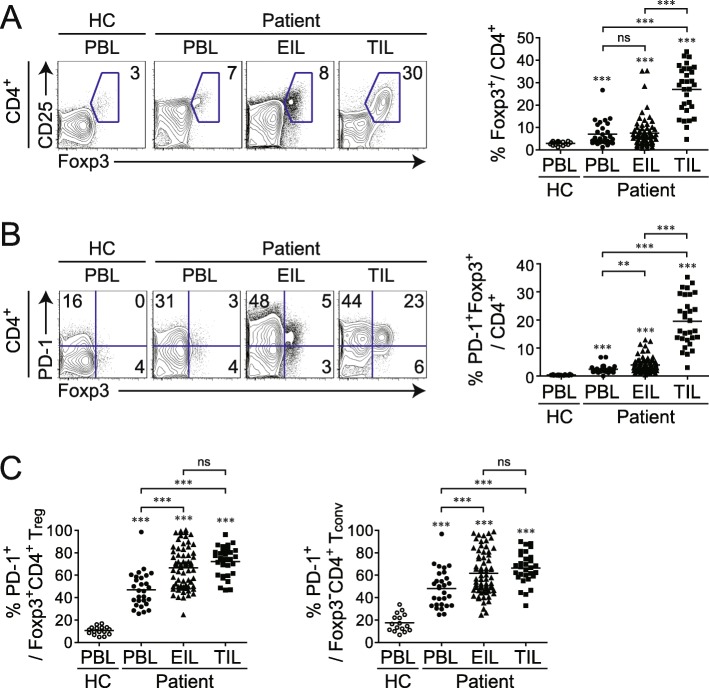
PD-1 expression in Foxp3^+^ T_reg_ in different tissue types of patients with cancer. **a** Representative plots of CD25 and Foxp3 expression (left) and proportion of Foxp3^+^ T cells (right) among CD4^+^ T cells. **b** Representative plots of PD-1 and Foxp3 expression (left) and proportion of PD-1 and Foxp3 co-expressing cells among total CD4^+^ T cells (right). **c** Summary of PD-1-positive fraction of Foxp3^+^ T_conv_ (left) and Foxp3^−^ T_reg_ (right) cell populations among CD4^+^ T cells. Peripheral blood lymphocytes (PBLs), effusion-infiltrating lymphocytes (EILs), and tumor-infiltrating lymphocytes (TILs) were isolated from healthy control donors (PBL, *n* = 16) and patients with cancer (PBL, *n* = 28; EIL, n = 76; TIL, *n* = 31). Lines in the scatterplot represent the mean values. ns, not significant; **P < 0.01, ***P < 0.001 (Mann-Whitney test)

We next examined how T_reg_ cells expressing forkhead box (Fox) p3 are distributed and differ phenotypically in PB, ME, and TMs. T_reg_ cells showed greater accumulation in TILs than in PBLs and EILs of patients or in PBLs of healthy controls (Fig. 2a). Interestingly, T_reg_ cells in TILs expressed a higher level of PD-1 than those in PBLs and EILs; moreover, the PD-1-expressing Foxp3^+^ population among CD4^+^ T cells was also larger in TILs than in EILs, which, in turn, had a larger population than PBLs (Fig. 2b). To further characterize CD4^+^ T cells in different tissues, we compared PD-1 in Foxp3^+^ and Foxp3^−^ CD4^+^ T cells (Fig. 2c). The proportion of PD-1-expressing cells in both CD4^+^ cells was larger in EILs and TILs than in PBLs. These results indicate that PD-1 expression by T_reg_ cells and T_conv_ cells clearly reflect the TME, as PD-1 expression increased in the following order: TILs > EILs > PBLs.

We next investigated whether the characteristics of CD4^+^ T cells, CD8^+^ T cells, and T_reg_ cells were altered in MEs depending on the cancer type. As shown in Additional file 1: Figure S1, the abundance of these cells and their expression of PD-1 in MEs were comparable in different types of cancer, although it is worth noting that there were more T_reg_ cells than there were CD4^+^ or CD8^+^ T_conv_ cells expressing PD-1. Interestingly, the degree of infiltration of PD-1^+^ T_reg_ cells did not differ among ME samples derived from the different types of cancer, indicating that the presence of PD-1^+^ T_reg_ cells in ME is a common feature across cancers of distinct histological origin (Additional file 1: Figure S1). Additionally, we compared the phenotype of T_reg_ between ascites and pleural effusion. As shown in Additional file 2: Figure S2, significant differences of the percentages of Foxp3^+^ T_reg_ and PD-1^+^ Foxp3^+^ T_reg_ cells were not observed between ascites and pleural effusion. Moreover, the ascites and pleural effusions had a similar expression rate of PD-1 in Foxp3^+^ T_reg_ and Foxp3^−^ T_conv_ cells.

### Tumor-infiltrating T_reg_ are abundant in patients with lung cancer and express multiple IC-molecules

**Fig. 3:**
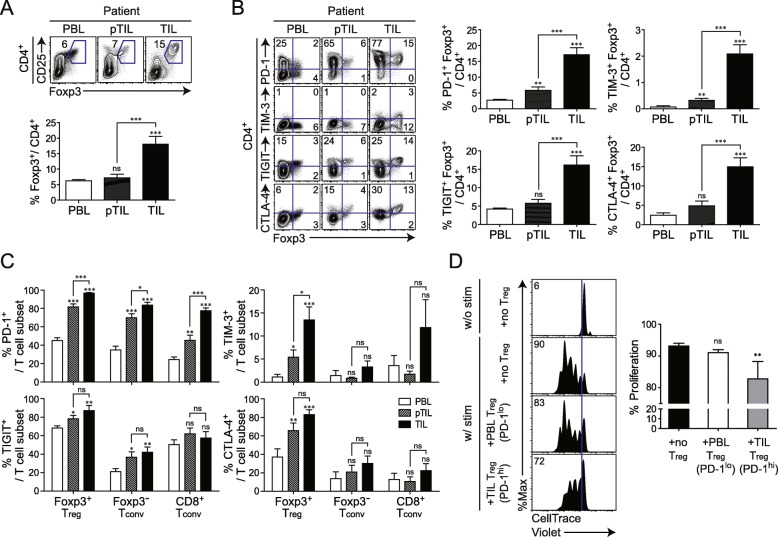
PD-1-expressing tumor-infiltrating T_reg_ and their activated phenotype in patients with non-small cell lung cancer (NSCLC). **a** CD25 and Foxp3 expression in CD4^+^ T cells (upper) and proportion of Foxp3^+^ cells among total CD4^+^ T cells (lower) in peripheral blood lymphocytes (PBLs), peritumoral infiltrating lymphocytes (pTILs), and tumor-infiltrating lymphocytes (TILs) derived from patients with NSCLC. **b** Representative plots of PD-1, TIM-3, TIGIT, CTLA-4, and Foxp3 expression in CD4^+^ T cells (left) and percentage of CD4^+^ T cells co-expressing PD-1, TIM-3, TIGIT, CTLA-4, and Foxp3 (right). **c** PD-1, TIM-3, TIGIT, and CTLA-4 expression in Foxp3^+^ T_reg_, Foxp3^−^ T_conv_ and CD8^+^ T_conv_ of these patients. **d** Enhanced suppression of CD8^+^ T cells by PD-1-expressing tumor-infiltrating T_reg_ from NSCLC patients. T_reg_ were isolated from the peripheral blood and tumor tissue from NSCLC patients. Peripheral blood T_reg_ and tumor-infiltrating T_reg_ expressed low and high levels of PD-1, respectively. CellTrace Violet (CTV)-labeled CD8^+^ T cells were stimulated in vitro with CD3/CD28 Dynabeads for 96 h in the absence or presence of each T_reg_ population. CTV dilution in proliferating CD8^+^ T cells is indicated in each histogram. Histograms represent the percentages of proliferating cells. Lines in the bar graph represent the mean and mean ± SEM, respectively. ns, not significant; **P < 0.01, ***P < 0.001 (Mann-Whitney test)

To clarify the characteristics of T_reg_ cells in the TME, we compared the frequency of T_reg_ cells and the expression of IC-molecules such as PD-1, TIM-3, TIGIT, and CTLA-4 in paired sets of tissue-derived lymphocytes, such as PBLs, pTILs, and TILs collected from 12 patients with NSCLC. As expected, T_reg_ cells were more highly enriched in TILs than in pTILs and PBLs (Fig. 3a). Moreover, more T_reg_-expressing ICs were found among TILs than among pTILs and PBLs (Fig. 3b).

We also compared IC-molecule expression on different tumor-infiltrating T cell subsets. Among four different IC-molecules, PD-1 most clearly distinguished TME in all T cell subsets, because a significant increase in the PD-1^+^ population was observed in the following order TIL > pTIL > PBL (Fig. 3c). Notably, PD-1 was higher in tumor-infiltrating Foxp3^+^T_reg_ cells (~ 98%) than in Foxp3^−^T_conv_ cells (~ 82%) or CD8^+^ T_conv_ cells (78%). Furthermore, the number of PD-1-expressing tumor-infiltrating Foxp3^+^ T_reg_ cells was greater than the number of tumor-infiltrating Foxp3^+^ T_reg_ cells expressing other IC-molecules. It is therefore conceivable that PD-1 expression on T_reg_ cells is a TME marker. Additionally, we performed the in vitro suppressive assay using isolated CD4^+^CD25^+^ T_reg_ cells from the peripheral blood and tumor tissue of NSCLC patients and isolated CD8^+^ T cells from the peripheral blood. Each tumor-infiltrating T_reg_ cells or peripheral T_reg_ cells was co-cultured with peripheral CD8^+^ T cells with αCD3/CD28 stimulation. CD8^+^ T cells proliferated at a high rate in the absence of T_reg_ cells and were more potently inhibited by PD-1^hi^ tumor-infiltrating T_reg_ cells than by PD-1^lo^ PBMC T_reg_ cells (Fig. 3d).

### T_reg_ numbers and the expression of IC-molecules are altered during cancer progression in a mouse model

**Fig. 4:**
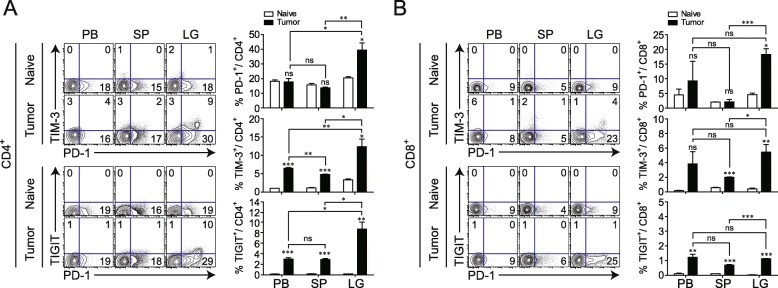
Differential expression of immune checkpoint (IC) molecules on CD4^+^ and CD8^+^ T cells in mice with lung cancer. To induce lung adenocarcinoma, TC-1 cells were intravenously injected into syngeneic mice. **a**, **b** Tumor-bearing mice at 3 weeks after TC-1 cell injection and naïve control mice were sacrificed, and lymphocytes were isolated from peripheral blood (PB), spleen (SP), and lung (LG). (Left) Expression levels of PD-1, TIM-3, and TIGIT on CD4^+^ and CD8^+^ T cells were assessed. (Right) Summary of the proportions of IC molecules expressed on populations of CD4^+^ and CD8^+^ T cells in PB, SP, and LG at the tumor site. Numbers in the plot indicate percentages of the corresponding population. Data are representative of three independent experiments (*n* = 5 mice per group in each experiment). ns, not significant; *P < 0.05, **P < 0.01, ***P < 0.001 (Student’s *t*-test). The symbol above each column is the *P* value obtained when SP samples were compared to the corresponding samples from naïve mice (control)

We previously showed that immune-exhaustion markers were highly expressed in tumor-infiltrating T_reg_ cells of patients with NSCLC. We therefore investigated the T_reg_ phenotype in greater detail in different tissues, using a mouse lung cancer model. We compared the expression levels of IC-molecules, such as PD-1, TIM-3, and TIGIT, on CD4^+^ and CD8^+^ T cells in different tissues from naïve and TM-bearing mice. As in patients with cancer tissue, the expression of IC-molecules in CD4^+^ and CD8^+^ T cells was much higher in lung TM than in PB or spleen (Fig. 4a, b). Among the populations expressing IC-molecules, PD-1-expressing CD4^+^ and CD8^+^ T cells were more abundant in the TM.

**Fig. 5:**
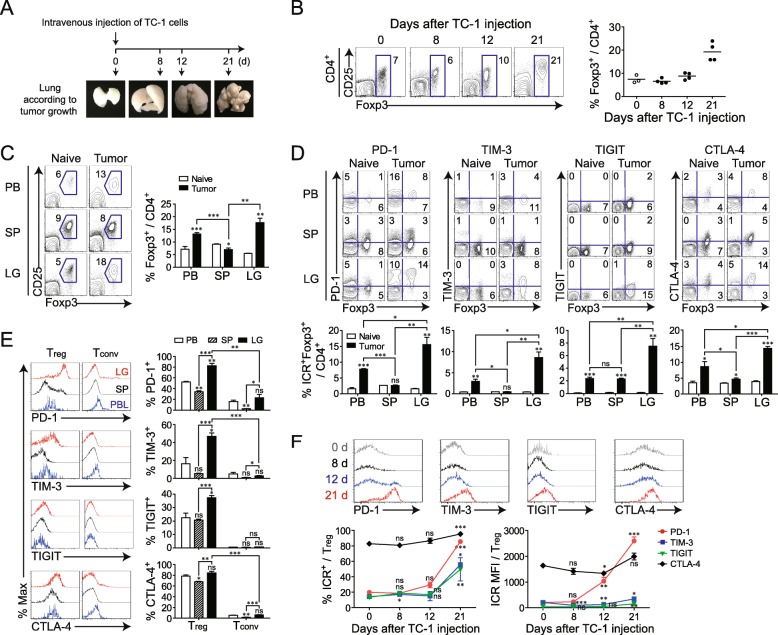
Spatial and temporal dynamics of immune checkpoint (IC) molecule expression on T_reg_ during cancer progression. **a** Schedule for establishing the TC-1 lung adenocarcinoma model and tumor formation at each time point. **b** Representative plots showing CD25 and Foxp3 expression in CD4^+^ T cells (left) and changes at different time points after TC-1 TM tumor cell injection (right). **c** Representative plots of T_reg_ (left) and summary of the proportion of Foxp3^+^ cells among CD4^+^ T cells (right) in peripheral blood (PB), spleen (SP), and lung (LG). **d** Levels of PD-1, TIM-3, TIGIT, and CTLA-4 expression on Foxp3^+^CD4^+^ T_reg_ in PB, SP, and LG. **e** Levels of PD-1, TIM-3, TIGIT, and CTLA-4 expression on T_reg_ and T_conv_ in different tissues (PB, SP, and LG). **f** Changes in the levels of PD-1, TIM-3, TIGIT, and CTLA-4 expression on T_reg_ at different time points. Data are representative of three independent experiments (*n* = 5 mice per group in each experiment). ns, not significant; *P < 0.05, **P < 0.01, ***P < 0.001 (Student’s *t*-test)

We next examined whether IC-molecules are preferentially upregulated on T_reg_ cells (compared to T_conv_) in TM, as was observed in patient tissues. PB, spleen, and lung lymphocytes were isolated at different time points after TC-1 injection (Fig. 5a). Starting at 12 days after TC-1 injection, an increase in the number of Foxp3^+^ T_reg_ cells was observed in TM and the T_reg_ cells fraction reached 20% of total CD4^+^ T cells, a nearly 3-fold increase compared to that in the non-TM lung (Fig. 5b). At 3 weeks after TC-1 injection, Foxp3^+^ T_reg_ cells were more abundant in the TM than in the PB or spleen (Fig. 5c). Foxp3^+^ T_reg_ cells in TM showed significant increases in PD-1, TIM-3, TIGIT, and CTLA-4, compared to other tissues (Fig. 5d). Moreover, tumor-infiltrating T_reg_ cells expressed much higher levels of IC-molecules than tumor-infiltrating T_conv_ (Fig. 5e). Most T_reg_ cells (~ 80%), but only a low frequency of T_conv_ (~ 20%) expressed PD-1 in TM. PD-1 was markedly upregulated 21 days after TC-1 injection, and the same trend was observed for TIM-3 and TIGIT, although the increases in the levels of these molecules were less prominent (Fig. 5f). Unlike PD-1, TIM-3, and TIGIT, CTLA-4 was already upregulated in T_reg_ cells before TC-1 injection and its expression progressively increased over time (Fig. 5f). Thus, expression of IC-molecules, especially PD-1, on T_reg_ cells increases with TM progression. As tumor numbers increased, immune checkpoints including PD-1, TIM-3, TIGIT, and CTLA-4 increased (Additional file 3: Figure S3).

### Immunosuppressive function of tumor-infiltrating T_reg_ in CD8^+^ T cell response is mediated by PD-1/PD-L1 interaction

**Fig. 6:**
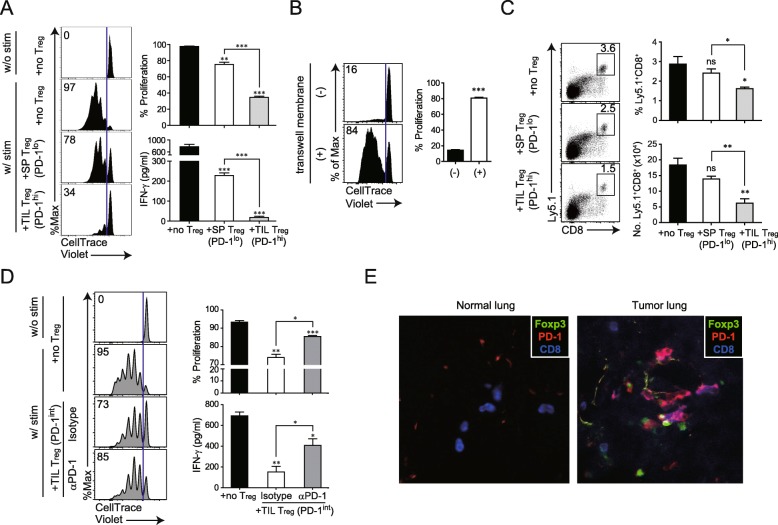
Enhanced suppressive function of PD-1-expressing tumor-infiltrating T_reg_. **a** Enhanced suppression of CD8^+^ T cells by PD-1-expressing tumor-infiltrating T_reg_. At 3 weeks after intravenous injection of TC-1 cells, T_reg_ were isolated from the spleen (SP) and lung of mice with TC-1 cell-induced tumors. SP T_reg_ and tumor-infiltrating T_reg_ expressed low and high levels of PD-1, respectively. CellTrace Violet (CTV)-labeled CD8^+^ T cells were stimulated in vitro with CD3/CD28 Dynabeads for 72 h in the absence or presence of each T_reg_ population. CTV dilution in proliferating CD8^+^ T cells is indicated in each histogram. Histograms represent the percentages of proliferating (upper) and IFN-γ-producing (lower) cells. **b** Contact-dependent T_reg_-mediated suppression of CD8^+^ T proliferation. CTV-labeled CD8^+^ T cells were stimulated in vitro with CD3/CD28 Dynabeads and cocultured with tumor-infiltrating T_reg_ for 72 h in the absence or presence of a transwell membrane. **c** Homeostatic proliferation of donor Ly5.1^+^CD8^+^ T cells in the spleen isolated from Rag2^−/−^ mice at 7 d after adoptive cell transfer. Representative plot (left) and absolute number (right) of donor Ly5.1^+^CD8^+^ T cells in the spleen. **d** PD-1-mediated suppressive activity of tumor-infiltrating T_reg_ isolated from the lungs of tumor-bearing mice 2 weeks after intravenous injection of TC-1 cells. At this time point, T_reg_ expressed intermediate levels of PD-1. CTV-labeled CD8^+^ T cells were stimulated as shown in (**a**). Before co-culture of CD8^+^ T cells with tumor-infiltrating T_reg_, the latter were pre-incubated with an anti-PD-1 antibody or its isotype as control. CTV dilution in proliferating CD8^+^ T cells is shown in the histograms, which represent the percentages of proliferating (upper) and IFN-γ-producing (lower) cells. (**e**) Representative immunofluorescence images of mouse lung tumor samples. Antibodies against Foxp3, CD8, and PD-1 were used to label and examine the interaction between T_reg_ and CD8^+^ T cells expressing PD-1. Data are representative of two independent experiments. *P < 0.05, **P < 0.01, ***P < 0.001 (Student’s *t*-test)

Among all IC-molecules examined, PD-1 was most highly upregulated in tumor-infiltrating T_reg_ cells. To determine the role of PD-1 on tumor-infiltrating T_reg_ cells, in the regulation of the CD8^+^ T cell response, we compared the suppressive activity of T_reg_ expressing high- and low-levels of PD-1 (PD-1^hi^ T_reg_ cells from lung TM 3 weeks after TC-1 injection vs. PD-1^lo^ T_reg_ cells from the spleen of the same TM-bearing mice). CD4^+^CD25^+^ T_reg_ cells, isolated using a microbead-based Treg isolation kit (CD4^+^CD25^+^ Regulatory T Cell Isolation kit), was confirmed to be ~ 90% purified Foxp3^+^ T_reg_ cells (Additional file 4: Figure S4). Each population was co-cultured with naïve CD8^+^ cells with or without stimulation by αCD3/CD28. CD8^+^ T cells proliferated at a high rate in the absence of T_reg_ cells and were more potently inhibited by PD-1^hi^ tumor-infiltrating T_reg_ cells than by PD-1^lo^spleen T_reg_ cells (Fig. 6a). Similarly, interferon (IFN)-γ production was also more strongly suppressed by PD-1^hi^ tumor-infiltrating T_reg_ than by PD-1^lo^ spleen T_reg_ cells.

To investigate the role of PD-1 upregulation, induced by tumor-infiltrating T_reg_ cells, we examined whether the interaction between PD-1 on tumor-infiltrating T_reg_ cells and PD-L1 on CD8^+^ T cells is required for immunosuppression in patients with cancer. PD-1 on tumor-infiltrating T_reg_ cells was blocked by incubation with an anti-PD-1 antibody. Unbound antibody was subsequently removed and the cells were co-cultured with CD8^+^ T cells. We prepared T_reg_ cells_,_ expressing an intermediate level of PD-1 that were isolated from lung TM in 2- rather than in 3-weeks after injection because T_reg_ cells_,_ highly expressing PD-1, isolated at later time-points, also co-expressed other IC-molecules (Fig. 5), making it difficult to differentiate the role of PD-1 in the suppressive function of T_reg_ cells from that of others.

Additionally, to clarify whether the potent suppressive function of PD-1^hi^ tumor-infiltrating T_reg_ cells is mediated via cell-to-cell contact between T_reg_ and CD8^+^ cells or soluble factors produced from T_reg_ cells, we conducted the experiments with transwell membrane system to block cell migration (Fig. 6b). Transwell membranes were inserted into 24-well plate. CTV labeled CD8^+^ T cells and CD4^+^CD25^+^ T_reg_ were placed into lower and upper wells, respectively, and αCD3/CD28 was added into both wells for stimulation. Suppression of T cell proliferation was not observed in the presence of the Transwell membrane. This data demonstrated that the suppression of CD8^+^ T cell proliferation by T_reg_ requires cell to cell contact between each cell population (Fig. 6b). Next, we performed the in vivo experiment with TIL T_reg_ and spleen T_reg_ cellsalong with Ly5.1^+^CD8^+^ T cells. In line with in vitro data, when TIL T_reg_ cells was injected, CD8^+^ T cell proliferation was significantly inhibited compared with spleen T_reg_ and no T_reg_ cells (Fig. 6c).

As shown in Fig. 6d, tumor-infiltrating T_reg_ cells that had been blocked with anti-PD-1 antibody, were significantly impaired in their ability to suppress the proliferation of CD8^+^ T cells and IFN-γ production as compared to isotype antibody-treated tumor-infiltrating T_reg_ cells. Given that both mouse and human CD8^+^ T cells can upregulate low affinity Fc receptors following activation [24, 25], we tested whether CD8^+^ T cells upregulate Fc receptors in our system. We obtained splenocytes of TC-1 tumor bearing mice. We stained splenocytes with CD8, CD44, CD16/32 (FcγRIII/II), and CellTrace Violet and compared the expression of Fc receptor between with and without stimulation. Significant differences of CD16/32 were not observed between groups with and without stimulation (Additional file 5: Figure S5A). To validate the CD16/32 antibody, we analyzed the expression of CD16/32 on NK cells and macrophages. This antibody can specifically detect CD16/32 on these cells, so we excluded the possibility that no detection of CD16/32 on CD8^+^ T cells after stimulation could be a problem of CD16/32 antibody (Additional file 5: Figure S5B). Taken together, our data demonstrated that the effect of anti-PD-1 is direct effect by blocking of PD-1 pathway signaling rather than the effect of anti-PD-1 antibody mediated by ADCC.

A multi-color immunofluorescence analysis revealed that CD8, PD-1, and Foxp3 were co-localized in mouse tumor tissues (Fig. 6e), implying that CD8^+^ cells and Foxp3^+^ T_reg_ cells spatially interact in the TME.

## Discussion

In this study, we examined the phenotype and function of T_reg_ cells as well as CD4^+^ and CD8^+^ T_conv_ cells that infiltrated into the TME, including the ME and TM from patients with cancer. We also investigated the mechanism by which T_reg_ cells induce immunosuppression using a mouse lung cancer model. Most tumor-infiltrating T_reg_ cells showed higher PD-1 expression than T_conv_ cells, implying that PD-1-expressing T_reg_ cells are a biological marker of the TME. Indeed, in T_reg_ cells derived from TMs of patients with NSCLC, PD-1 was the most clearly upregulated IC-molecule. As previously reported, these cells exhibited an enhanced immunosuppressive function that was correlated with the extent of PD-1 upregulation [12]. We speculate that PD-1-expressing tumor-infiltrating T_reg_ cells induce immunosuppression through the interaction of PD-1 and PD-L1, which may contribute to immune escape in TME. Clarifying the link between this phenotype and enhanced suppressive function of tumor-infiltrating T_reg_ cells can provide insight into their suppressive mechanism in patients with cancer.

The predominant function of PD-1 in T_reg_ cells seems to be similar to that of CTLA-4; both proteins contribute to the maintenance of T_reg_ immunosuppressive function [15]. However, PD-1 expression on T_reg_ cells differed by cell location. For instance, PD-1 was expressed by T_reg_ cells in TMs but not in normal tissue or PBLs as depicted in Fig. 3. In contrast, T_reg_ cells had high basal CTLA-4 levels irrespective of the tissue of origin. This supports our assertion that PD-1 on T_reg_ cells is a more useful marker for characterizing the TME. We also examined whether the upregulation of PD-1 on tumor-infiltrating T_reg_ cells can reinforce their basal immune-suppressive function. High PD-1 expression in T_reg_ cells was associated with the suppression of CD8^+^ T cells and PD-1 blockade abrogated the immune-suppressive function of T_reg_ cells, suggesting that an interaction between PD-1 on T_reg_ cells and PD-L1 expressed by another cell type—likely CD8^+^ T cells [26]—is necessary for immunosuppression. Thus, elevated PD-1 expression on T_reg_ cells is a potential marker for immune escape in patients with cancer. These findings were consistent with our previously reported data that PD-1 upregulation in T_reg_ cells and the interaction between PD-1 on T_reg_ cells and PD-L1 expressed by effector T cells enhanced T cell-mediated immune suppression during chronic viral infection [12]. Thus, an immunotherapy targeting T_reg_ and PD-1 could be highly effective in patients with cancer.

We also investigated tumor-infiltrating T_reg_ and T_conv_ cells obtained from ME of patients with stage IV cancer. Most of the earlier studies of TME T_reg_ cells were performed in mice and focused on T_reg_ cells phenotype. Studies in patients with stage IV cancer have been hampered by the difficulty of obtaining sufficient TMs for analysis of T cell populations. To overcome this limitation, we developed an experimental model using ME from human patients with stage IV cancer as representative TME of stage IV cancer. This model will allow future examinations of various mechanistic aspects of human cancer through functional assays.

Several studies have reported IC expression on intra-tumoral T_reg_ cells and suggested potential roles of these ICs in the regulation of the immune response in mice [6, 15, 27]. We also showed here that ICs other than PD-1 were upregulated in T_reg_ cells. Studies on the relative contributions of these IC-molecules to immunosuppression in the TME may lead to the development more effective immunotherapies.

Regarding other PD-1-expressing immune cells than CD8^+^ T cells and T_reg_ cells in TME and their role, Irving et al. reported that tumor-associated macrophages (TAMs) expressed PD-1 and PD-1-expressing TAMs increased over time in mouse model and progressive disease in human cancers [28]. PD-1 expressed on TAMs reduced their phagocytic potency against tumor cells and blockade of PD-1 pathway restored the macrophage phagocytosis, resulting in enhancing anti-tumor activity of TAMs. This data suggests that PD-1 expressed by TAMs is one of the mechanism for immune evasion. PD-1 expression was also described on NK cells in many different types of human and mouse cancers, where the PD-1 expressed by NK cells negatively regulated NK cell function even though its molecular mechanisms are not clearly demonstrated to date [29–34]. In addition, PD-1 has been reported to be expressed on innate lymphoid cells (ILCs), prevalently ILCs type 3 (ILC3s), as well as NK cells in pleural effusion of primary and metastatic tumors, albeit the role of PD-1 on ILC3s was not addressed [35].

Based on these reports, it is plausible that PD-1 expressed by different types of immune cells including CD8+ T cells, T_reg_ cells, NK cells, and ILCs in the TME probably contributes to immune evasion, leading to promotion of tumor cells. However, it has not been addressed yet which types of PD-1-expressing immune cells are most effectively involved in the PD-1-mediated immunosuppression. In addition, to compare the immunosuppressive activity of each immune cell subset, the level of PD-1 expression on each type of cells should be examined. In this regard, further study is needed to determine whether other PD-1-expressing immune cells than T_reg_ cells in the TME compensate for a lack of T_reg_ cells and which types of PD-1-expressing immune cells mostly impact on immune suppression in the TME.

T_reg_ cells expansion in the TME is widely recognized as an obstacle to successful immunotherapy in patients with cancer [5]. Previously, we demonstrated that T_reg_ cells depletion using an anti-CD25 antibody increased the abundance of functional antigen-specific CD8^+^ T cells during chronic viral infection [12]. Furthermore, treatment with a neutralizing antibody also caused the elimination of non-T_reg_ and rapid replenishment of T_reg_ cells [36]. Thus, functional inactivation of T_reg_ cells and rejuvenation of exhausted T cells by targeting overexpressed PD-1 combined with temporal depletion of T_reg_ cells expressing IC-molecules may be a promising strategy to limit cancer progression.

## Conclusions

In conclusion, our study provides insights into T_reg_ cells function and their suppressive mechanism in patients with cancer. We showed that the suppressive function of tumor-infiltrating T_reg_ cells was enhanced by the increase in their relative proportion and by the upregulation of the expression of inhibitory receptors, such as PD-1, TIM-3, and CTLA-4.

## Supplementary information

**Additional file 1: Figure S1**. PD-1 expression on CD4^+^, CD8^+^, or T_reg_ in malignant effusion according to the different types of cancer patients including lung cancer, gastric cancer, breast cancer, and others.

**Additional file 2: Figure S2**. Comparison of T_reg_ phenotype between ascites and pleural effusion. (A) Percentage of Foxp3 in total CD4^+^ T cells between ascites and pleural effusion (B) Percentage of PD-1^+^Foxp3^+^ in total CD4^+^ T cells between ascites and pleural effusion (C) Percentage of PD-1^+^ in Foxp3^+^CD4 T_reg_ between ascites and pleural effusion (Left), percentage of PD-1^+^in Foxp3^−^ CD4 T cells between ascites and pleural effusion.

**Additional file 3: Figure S3**. Correlation of the immune checkpoint including PD-1, TIM-3, TIGIT and CTLA4 expressed on T_reg_ as tumor nodule number increased. (A) PD-1, (B) TIM-3, (C) TIGIT, and (D) CTLA-4 expression on T_reg_ according to the increased tumor nodules. The number of tumor nodules was measured at day 12, 16, and 21 post-injection (*n* = 4–7 mice per group). Data are representative of two independent experiments. *P < 0.05, **P < 0.01, ***P < 0.001 (Student’s *t*-test).

**Additional file 4: Figure S4**. Purification of tumor-infiltrating T_reg_ using microbead-based Treg isolation kit. T_reg_ were separately isolated from the spleen and from TM-bearing mice using a CD4^+^CD25^+^ Regulatory T Cell Isolation kit for suppressive function analysis. T_reg_, isolated using a microbead-based Treg isolation kit, demonstrated ~ 90% purified Foxp3^+^ T_reg_ compared with the 16% prior to isolation.

**Additional file 5: Figure S5**. Expression of CD16/32 on CD8^+^ T cells after in vitro TCR activation and NK cells and macrophages. (A) The expression of CD16/32 on purified CD8^+^ T cells activated by CD3/28 Dynabeads for 3 d. (B) The expression of CD16/32 on Dx5^+^NK1.1^+^ NK cells and CD11b^+^F4/80^+^ macrophages isolated from the spleen of naïve mouse.

**Additional file 6: Table S1.** Baseline characteristics of patients and specimens.

## Data Availability

All data generated or analysed during this study are included in this published article and its supplementary information files.
