# Supplementary figures and images for: Tumor microenvironment dictates regulatory T cell phenotype: Upregulated immune checkpoints reinforce suppressive function
Source: J Immunother Cancer. 2019 Dec 4;7:339. doi: 10.1186/s40425-019-0785-8 (PMC6894345; doi:10.1186/s40425-019-0785-8)

A

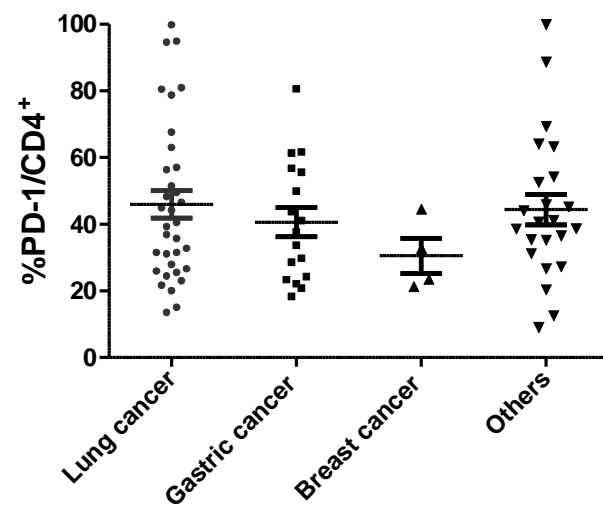

B

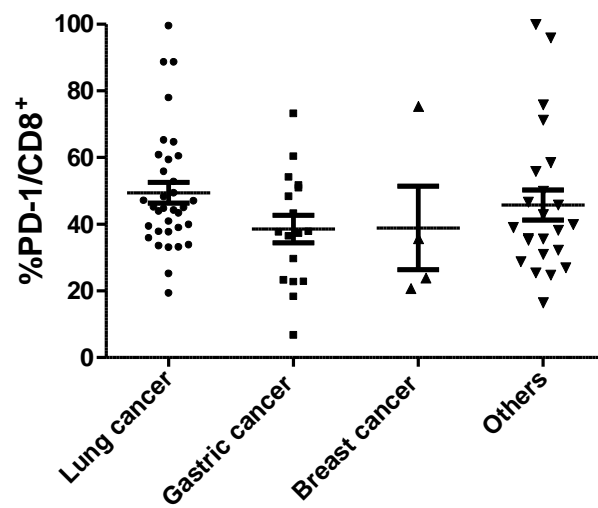

C

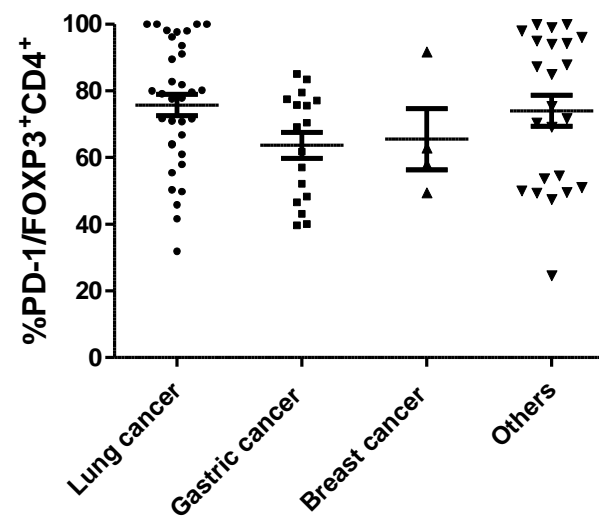

Supplement: Supplementary file 1 — Additional file 1: Figure S1. PD-1 expression on CD4+, CD8+, or Treg in malignant effusion according to the different types of cancer patients including lung cancer, gastric cancer, breast cancer, and others. [file 40425_2019_785_MOESM1_ESM.pdf]

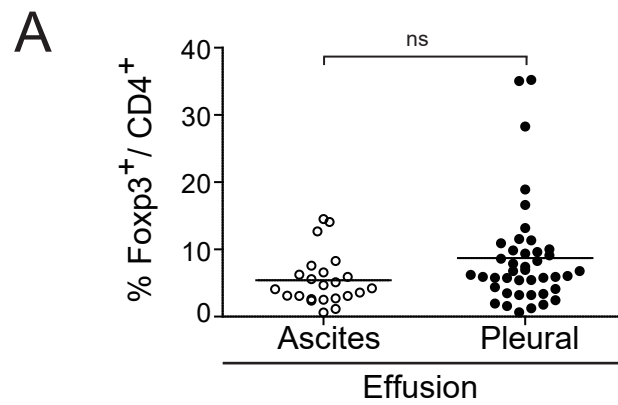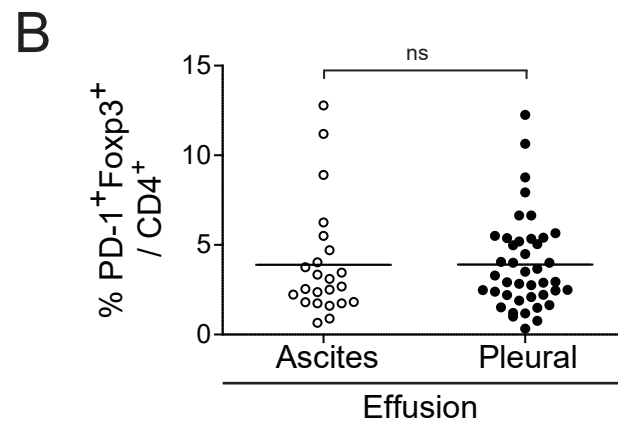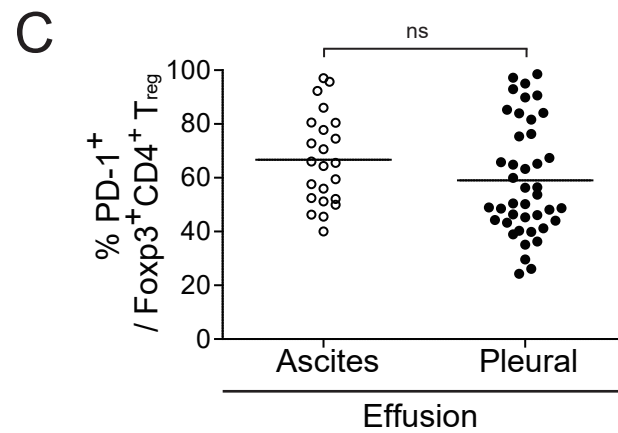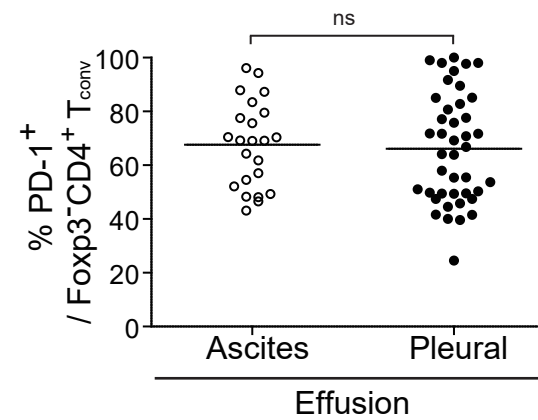

Supplement: Supplementary file 2 — Additional file 2: Figure S2. Comparison of Treg phenotype between ascites and pleural effusion. (A) Percentage of Foxp3 in total CD4+ T cells between ascites and pleural effusion (B) Percentage of PD-1+Foxp3+ in total CD4+ T cells between ascites and pleural effusion (C) Percentage of PD-1+ in Foxp3+CD4 Treg between ascites and pleural effusion (Left), percentage of PD-1+in Foxp3− CD4 T cells between ascites and pleural effusion. [file 40425_2019_785_MOESM2_ESM.pdf]

A

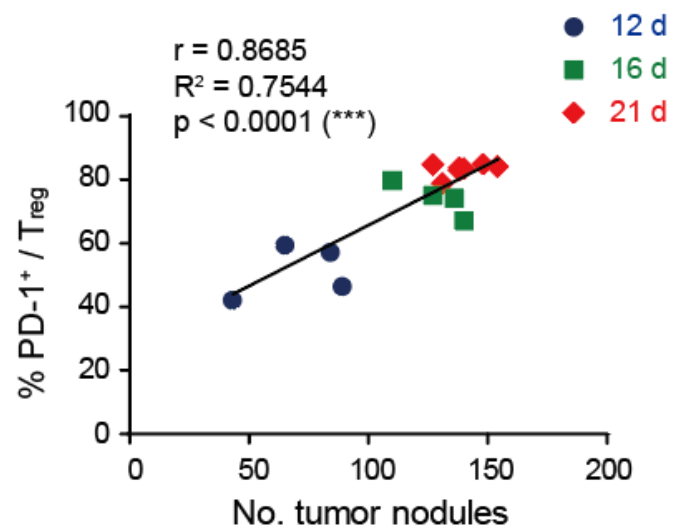

B

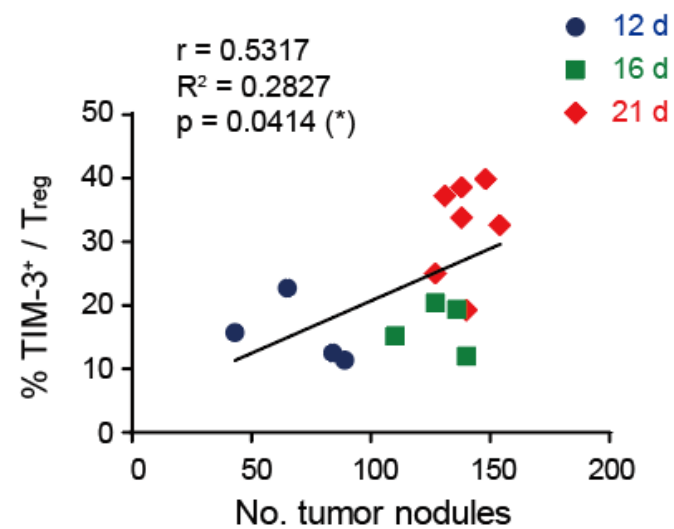

C

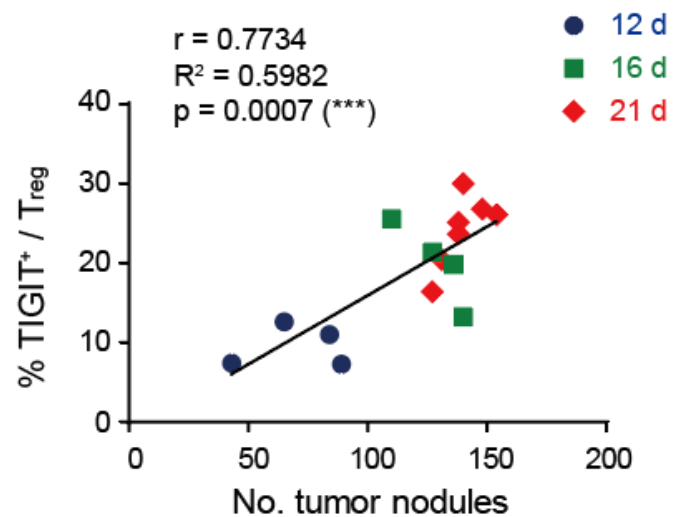

D

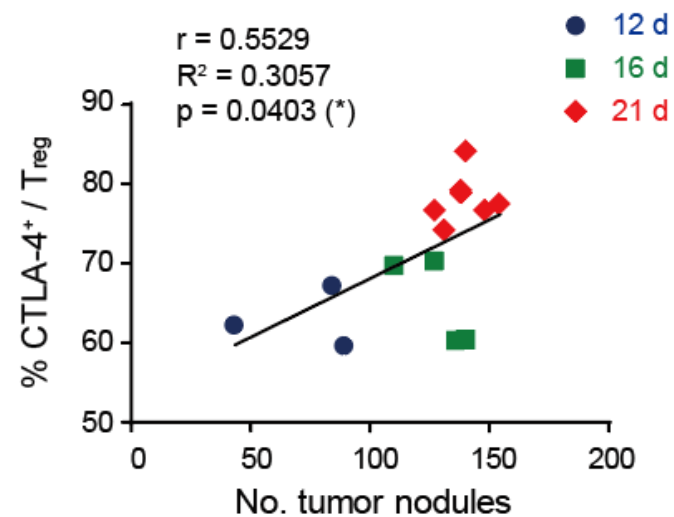

Supplement: Supplementary file 3 — Additional file 3: Figure S3. Correlation of the immune checkpoint including PD-1, TIM-3, TIGIT and CTLA4 expressed on Treg as tumor nodule number increased. (A) PD-1, (B) TIM-3, (C) TIGIT, and (D) CTLA-4 expression on Treg according to the increased tumor nodules. The number of tumor nodules was measured at day 12, 16, and 21 post-injection (n = 4–7 mice per group). Data are representative of two independent experiments. *P < 0.05, **P < 0.01, ***P < 0.001 (Student’s t-test). [file 40425_2019_785_MOESM3_ESM.pdf]

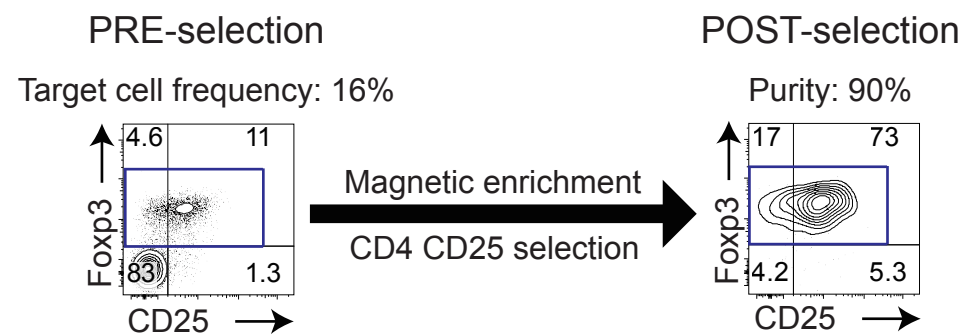

Supplement: Supplementary file 4 — Additional file 4: Figure S4. Purification of tumor-infiltrating Treg using microbead-based Treg isolation kit. Treg were separately isolated from the spleen and from TM-bearing mice using a CD4+CD25+ Regulatory T Cell Isolation kit for suppressive function analysis. Treg, isolated using a microbead-based Treg isolation kit, demonstrated ~ 90% purified Foxp3+ Treg compared with the 16% prior to isolation. [file 40425_2019_785_MOESM4_ESM.pdf]

Figure S5

A

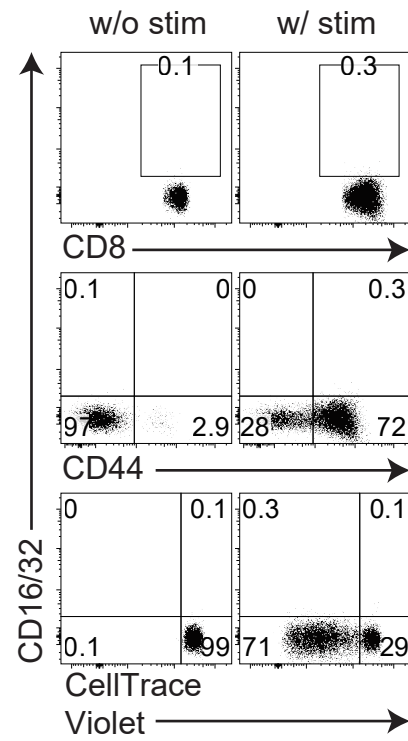

B

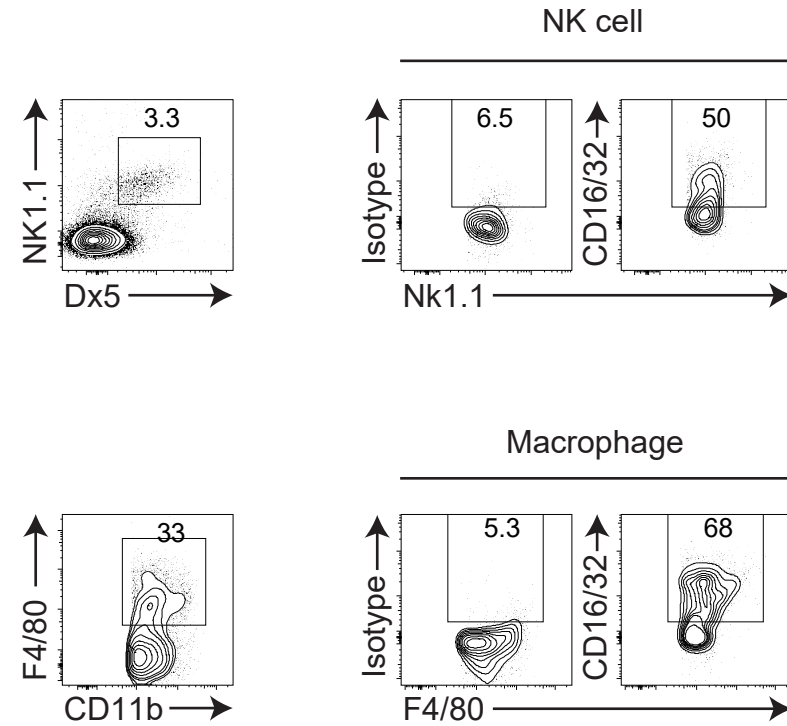

Supplement: Supplementary file 5 — Additional file 5: Figure S5. Expression of CD16/32 on CD8+ T cells after in vitro TCR activation and NK cells and macrophages. (A) The expression of CD16/32 on purified CD8+ T cells activated by CD3/28 Dynabeads for 3 d. (B) The expression of CD16/32 on Dx5+NK1.1+ NK cells and CD11b+F4/80+ macrophages isolated from the spleen of naïve mouse. [file 40425_2019_785_MOESM5_ESM.pdf]
