# Supplementary material for: Tumor microenvironment dictates regulatory T cell phenotype: Upregulated immune checkpoints reinforce suppressive function
Source: J Immunother Cancer. 2019 Dec 4;7:339. doi: 10.1186/s40425-019-0785-8 (PMC6894345; doi:10.1186/s40425-019-0785-8)
Supplement: Supplementary file 6 — Additional file 6: Table S1. Baseline characteristics of patients and specimens. [file 40425_2019_785_MOESM6_ESM.docx]

| **Supplementary Table 1.** Baseline characteristics of patients and specimens | | | | |
| --- | --- | --- | --- | --- |
| Characteristic | Total patient | Group | | |
|  |  | Peripheral blood  (PBL) | Malignant effusion  (ME) | Tumor  (TM) |
| Number of patients | 103 | 28 (27.1%) | 72 (69.9%) | 31 (30.0%) |
| Number of specimens | 135 | 28 (20.7%) | 76 (56.2%) | 31 (23.1%) |
| Age in years (median, range) | 62 (33–83) | 62 (35–81) | 61 (33–83) | 60 (34–76) |
| Sex |  |  |  |  |
| Male | 50 (48.5%) | 16 (57.1%) | 32 (44.4%) | 18 (58.1%) |
| Female | 53 (51.5%) | 12 (42.9%) | 40 (55.6%) | 13 (41.9%) |
| Cancer type |  |  |  |  |
| Non-small cell lung cancer | 37 (35.9%) | 14 (50%) | 31 (43.1%) | 6 (19.4%) |
| Gastric cancer | 16 (15.5%) | 7 (25%) | 16 (22.2%) | 0 (0%) |
| Colon cancer | 29 (28.2%) | 1 (3.6%) | 4 (5.6%) | 25 (80.6%) |
| Breast cancer | 4 (3.9%) | 1 (3.6%) | 4 (5.6%) | 0 (0%) |
| Others | 17 (16.5%) | 5 (17.8%) | 17 (23.6%) | 0 (0%) |
| Number of samples |  |  |  |  |
| Peripheral blood | 28 (20.7%) | 28 (100%) | − | − |
| Malignant pleural effusion* | 33 (24.4%) | − | 33 (59.7%) | − |
| Malignant ascites* | 43 (31.8%) | − | 43 (45.8%) | − |
| Tumor tissue | 31 (23.1%) | − | − | 31 (100%) |

* Four patients having both pleural effusion and ascites.
